# Are Front-of-Pack Nutrition Labels Influencing Food Choices and Purchases, Diet Quality, and Modeled Health Outcomes? A Narrative Review of Four Systems

**DOI:** 10.3390/nu15010205

**Published:** 2023-01-01

**Authors:** Véronique Braesco, Adam Drewnowski

**Affiliations:** 1 VAB-Nutrition, 63100 Clermont-Ferrand, France; 2 Center for Public Health Nutrition, University of Washington, Seattle, WA 98195, USA

**Keywords:** Nutri-Score, Health Star Rating, traffic lights, warning signs, food reformulation, nutrient intakes

## Abstract

Front-of-Pack Nutrition labels (FOPNLs) aim to improve consumers’ food purchases and prompt product reformulation by the food and beverage industry. Despite their widespread use, the effectiveness of FOPNL in achieving these goals is still a matter of debate. This review has gathered 65 original studies exploring the performances of four widely used FOPNLs (Multiple Traffic Light, Warning signs, Nutri-Score and Health Star Rating). Although FOPNLs have been associated with healthier food purchases, the magnitude of improvements was small and dependent on study settings. Any associated health effects were modeled rather than observed. None of the four FOPNLs clearly outperformed the other ones on any outcome. Few studies dealt with the impact of FOPNL on product reformulation. Some of those studies, but not all, found small reductions in energy, sodium, sugar and saturated fat content of foods in some food categories. Although global trends point to a small favorable effect of FOPNL, this conclusion is subject to caution since the evidence is inconsistent and comes from a wide variety of contexts and study designs. There remain numerous research gaps, notably with regard to the optimal characteristics of FOPNLs, the durability of FOPNL effects on consumer behaviors, and any possible unexpected consequences.

## 1. Introduction

Front-of-Pack nutrition labels (FOPNLs) are increasingly displayed on packaged foods, worldwide. The first goal of FOPNLs is to provide nutrition information about a food at a glance, without the need to look at the more detailed back-of-pack nutrition facts panel. The relevant nutrition information may vary by region, depending on the dietary and health concerns of a given population. In many countries, FOPNLs are often meant to alert consumers to products that contain excessive amounts of energy, sugars, (saturated) fats and sodium. Fewer FOPNLs help consumers identify foods that include positive nutrients or under-consumed food groups.

There are two major categories of FOPNL symbols [1,2]. First, nutrient- specific or fact-based—FOPNLs provide information on selected nutrients of public health concern; these FOPNLs can be quantitative, evaluative or both; they are often derived from “guideline daily amounts” which were initially neutral (no color code) but later turned into traffic lights (green, amber, red) or into black Warning Signs (WS). Evaluative summary systems, the second category, are based on nutrient profile models that are intended to capture overall nutritional value, based on energy content and nutrient composition of foods. For a given food, an algorithm-derived evaluative score is then translated into a symbol, a numerical score or a letter grade that is intended to convey the overall healthfulness of the food to the consumer.

FOPNLs are typically implemented to help fulfill several public health objectives. A major goal is to guide consumers towards healthier food choices and thus improve their overall diet quality, leading to better health outcomes. In this respect, the World Health Organization recommends that governments implement FOPNLs, as part of a comprehensive policy to promote healthy diets [3].

FOPNLs have also been used to regulate marketing and advertising to children, to decide on health and nutrition claims or to justify implementing taxes on certain foods. FOPNL can also be used to encourage product reformulation, a health priority for many national governments and international agencies [4,5].

Countless FOPNLs schemes have been proposed, by various stakeholders, but only around 30 are today endorsed by governments, worldwide, with around ten more countries considering such endorsement [3]. All continents are concerned, with an early interest shown in Europe (Nordic keyhole, Choice program, Traffic light, Heart symbol, Nutri-Score, etc.) soon joined by Latin America (Warning Signs) and several Asian countries, including South Korea (Traffic lights), Thailand or Singapore (healthier choice logo). Middle East countries also support FOPNL, such as Iran (Traffic Light labels), United Arab Emirates (Weqaya logo) or Israel (green signpost and red warning labels), without forgetting the long-lasting commitment of Australia and New-Zealand, through the Health Star rating. Less initiative are seen in Africa, although Nigeria implemented the “Heart tick” and Zambia, the “Good Food logo” [3].

FOPNLs, or can be strongly recommended or even mandated by governmental authorities. The regulatory status of FOPNLs varies according to countries. In some instances, the law makes a FOPNL mandatory, such as in Chile [6]. In other geographies, FOPNLs can be used voluntarily by food manufacturers. This is the case in the European Union, where the regulation today forbids mandatory FOPNL [7], although the “Farm-to-Fork” policy has committed to modify this, and to implement a mandatory harmonized FOPNL by the end of 2022 [8]. However, no decision has been taken today and this commitment may be postponed, leaving European consumers with a very fragmented labelling landscape.

Despite a growing number of studies, the extent to which FOPNLs can support public health goals is still a matter of debate. In this review, we gathered studies addressing four major FOPNLs and attempted to analyze the direct impact of FOPNLs on food purchases, consumption patterns, and where possible health outcomes. We also examined the impact of common FOPNL as incentives for the food industry to reformulate and improve the nutrient density of their products.

## 2. Materials and Methods

### 2.1. Scope of the Review

Four FOPNLs were selected for this review: Multiple Traffic Light (MTL), Warning signs (WS), the Nutri-Score and the Health Star rating (HSR). Several criteria guided this selection, and primarily the availability of scientific studies on these FOPNLs. Of importance also was their endorsement by one or several governments, enabling a significant use in real life and thus possibilities to address the impact on the quality of the food products. Finally, a large geographic scope and some representativeness of the different possible schemes were considered. Regarding the latest, MTL (from UK) and WS (from Latin America) are “nutrient-specific” indicators, whereas Nutri-Score (Europe) and HSR (Australia & New Zealand) are summary indices of nutritional value. All can be qualified as “evaluative”, i.e., they “*provide interpretation across the levels of healthfulness for multiple product options. […] they can help consumers interpret the meaning of specific nutrient levels, as well as a product’s overall healthfulness*” [9]. Table 1 describes the key features of these four FOPNLs, together with data on current use of each FOPNL in their respective geographies.

This review has focused on three specific outcomes, thought to be the most directly related to FOPNL performance and of most interest to policy makers, regulators, and consumers Two of these were related to the impact of FOPNL on consumer behavior. The first one addressed whether and how the display of FOPNL could impact on the selection or purchase of foods. The second dealt with, nutrient intakes, quality of the overall diet and any potential health outcomes such as body weight, non-communicable disease risk and associated mortality. Of note, consumers’ perception, liking or understanding of the FOPNL were not considered, as they do not inform about the actual efficacy of the FOPNL. The third outcome concerned the impact of FOPNL on reformulation initiatives by the food industry and the nutritional quality of the packaged foods that may result from the removal/reformulation of existing products, or the launch of new ones.

### 2.2. Literature Search and Analysis

The scientific literature pertaining to these four FOPNL and three outcomes was searched from July 2018 up to end of May 2022. A prior systematic and comprehensive review of “Front-of-pack nutrition labelling schemes” has been produced by the Joint Research Centre (JRC) of the European Commission (EC) [18], covering the period up to end of June 2018. That report addressed *inter alia* the FOPNL and outcomes selected in the present review. The conclusions of JRC report were taken as a foundation for the present review, which was enriched by the studies published since July 2018.

The search was performed on Scopus for studies featuring the keyword “front of pack labelling” in various spellings in their title or abstract. Identified studies were then manually selected such as to retrieve original experimental or observational studies that dealt with at least one of the selected FOPNL and one of the selected outcomes. Modelling studies extrapolating from experimental or observational findings were also included.

Information was extracted from each study in an Excel file which featured the name of FOPNL(s) and outcome(s) addressed in the study, details on the publication (year, reference), population (country, sample size, age, gender, other), study methodology (objective, setting, design, food groups/categories when relevant, outcomes, statistics). Study results were summarized in a way that indicated whether the considered FOPNL was efficient, partially efficient, or non-efficient vs the considered outcome (Supplementary Table S1).

## 3. Results

The selection process retrieved a total of 64 original publications, that reported 65 experimental studies, addressing the selected FOPNL and selected outcomes, as displayed on Table 2 [12,19,20,21,22,23,24,25,26,27,28,29,30,31,32,33,34,35,36,37,38,39,40,41,42,43,44,45,46,47,48,49,50,51,52,53,54,55,56,57,58,59,60,61,62,63,64,65,66,67,68,69,70,71,72,73,74,75,76,77,78,79,80,81]. Details about each study are available in the Supplementary Table.

Studies have been mostly carried out in geographies where the selected FOPNL come from or are currently in use or debated. Europe is widely represented (15 studies in France, seven in Spain, five in the UK and in Belgium, four in Denmark and in the Netherlands, three in Portugal, two in Greece and one in nine other countries), as well as Oceania (ten studies in Australia and four in New Zealand) and, to a lower extent in Latin America (four studies in Uruguay and in Chile, three in Brazil, two in Peru and one in four other countries). Fewer studies came from North America (three from Canada and a single one in the USA), Asia (two studies in Singapore) and Africa (one study from Morocco), which is not surprising given the lower incidence of FOPNL overall in these regions.

In terms of study design, 45 studies were experimental with a single assessment of the outcome (among which 32 were performed online and 13 in a lab setting), four were experimental with long term assessment of the outcome (one was performed online and three were in a real-life setting (supermarket or catering)). Six studies were observational (one was cross-sectional and five were longitudinal,). Six were modeling studies and 4 were interrupted time series, combining two cross-sectional studies conducted at a few years interval.

### 3.1. The Influence of FOPNLs in Prompting Healthier Food and Beverage Choices

Studies addressing this outcome have attempted to follow consumer behavior either when they really purchased foods, in a real, on-line, or experimental supermarket (i.e., paying the food with their own money) or when they selected the foods they would have bought if they had been in a purchasing situation.

Conclusions of the JRC report from the few earlier (i.e., before mid-2018) publications on this topic distinguished studies according to their settings. The results from three laboratory experiments suggested that “colour-coded FOP schemes (Multiple Traffic Lights, NS) serve consumers best in making more nutritious food purchases” [15]. When considering real-life studies (five studies were identified that considered a FOPNL of interest in this review), the evidence suggested that “the impact of FOPNL on the healthfulness of food purchases was small, even if statistically significant at times” [18].

When considering studies published from mid-2018, 47 new set of data were identified and analyzed [12,20,23,24,27,29,30,32,33,34,35,36,37,38,39,40,41,42,44,46,49,50,51,52,53,55,56,58,59,60,62,63,64,65,66,67,68,69,70,71,72,73,74,78,79,81]. Among those, 23 compared two of the four FOPNL of interest or more. Most studies found a favorable impact of at least one FOPNL on at least one measure and only five studies (11%) did not find any effect on selection/purchasing behavior [12,38,39,52,63,71]. Conversely, several comparative studies reported a lack of effect of at least one FOPNL, even though another one was found effective. For example, a study in Portugal assessed the association between FOPNLs and the nutritional quality of choices of pizza, cakes, and breakfast cereals; MTL, Nutri-Score and WS were more helpful in driving consumers to healthier choices than neutral nutrition information (reference intakes), but HSR was not [66]. Furthermore, studies’ results often differed across different types of food products or food categories. In the above quoted study, the MTL led to healthier selection of cakes, but did not improve the selection of pizza or breakfast cereals [66]. Of note also, the favorable effects of FOPNLs on food choices is often of limited magnitude such as in a study which monitored the effects of Nutri-Score, MTL, HSR, WS and a non-interpretative label (reference intakes) on the choices made by subjects from 12 countries worldwide among nine products (three cakes, three pizza and three breakfast cereals) of high, intermediate, or low nutritional quality. For the vast majority of choice (82%), food selection remained unchanged from the non-labelled to the labelled condition. Improvements in the healthiness of products chosen occurred only for 12% of choices and a deterioration occurred for 6% [35]. It should be pointed out that most studies focused on a limited number of product categories, and may thus have missed some cross category shifts. A broad vision of FOPNLs effect on the whole food supply is still missing. Further, the effect of FOPNL on food selection/purchase was highly dependent on study setting.

#### 3.1.1. Findings from Laboratory-Based Experimental Studies

A large majority of the 45 newly identified studies were carried out in laboratory-type experimental settings and included volunteer participants who either came to a specific facility, such as an experimental market place or were connected to a specific online interface. These artificial environments provide standardized conditions and, especially when participants are randomized across conditions, decrease the impact of potentially confounding factors. However, these studies “examined intention to purchase rather than actual purchasing behavior, [and report] choice that has limited external validity”, according to [18]. Among the 40 studies that used such experimental environment, all but five [12,38,52,63,71] concluded to a favorable effect of FOPNL on the healthiness of food selection/purchase. In a French study, participants were randomized to shop twice from an online catalog of 290 food products that either displayed no FOPNL or one of five FOPNL, including Nutri-Score and HSR. The nutritional quality of the basket was evaluated using the score provided by the FSA nutrient profiling model [82] which spreads over 55 points (−15 to +40). Compared to the situation without a FOPNL, the nutrition score of the food was improved by 2.65 and 1.86 points when Nutri-Score and HSR, respectively, were displayed [56]. However, the FSA model used to assess the nutritional quality of foods was basically the same as the algorithm that supports Nutri-Score and closely linked to the HSR-supporting algorithm, meaning that for those two systems the exposure and the outcome variables were calculated in a very similar way. Not surprisingly Nutri-Score and HSR performed better than the other FOPNL schemes.

#### 3.1.2. Findings from Real-Life Supermarket Studies

Four empirical “real-life” studies were performed in usual shopping situations [12,28,33,75], and one in a real catering situation [69]; they enrolled subjects who were hardly or not aware they were participating in a study. Such studies are more realistic and their findings are easier to generalize, but they may be affected by confounding factors such as brand loyalty, habits, promotion and marketing, or seasonality.

Two studies exploited purchase data from Kantar consumers’ panels: one followed purchases of carbonated soft drinks by 1646 Ecuadorian households for 20 months before and 16 months after the implementation of MTL. No significant improvement was observed in purchases of highly sweetened drinks after MTL have become mandatory [12]. A different conclusion was reached when observing the quality of the food basket before and after implementation of various (not individualized in study results) FOPNLs by British retailers on seven food categories. It seems that purchase behavior of 20,707 households have changed towards healthier food selections, following displaying of FOPNL. However, this result may have been confounded by many other factors: during the three-year period of survey, it is likely that opinions and sensitivity around nutrition and food supply have evolved in UK due, for example, to prevention or marketing communication campaigns [33].

A third real-life study was carried out in Belgian supermarkets without, then with, the display of a black-and-white version of the NS on electronic shelf labels (intervention supermarkets). Purchases in control supermarkets (no FONL ever displayed) were also followed, which reduced the bias due to period-related confounders. Results were mitigated and of limited magnitude, with a favorable effect of NS found for products bearing a B and C letter (good to intermediate nutritional quality), unfavorable effects for products bearing a D letter and no effect for on products labeled with an A or E (best and worst nutritional quality). Authors concluded that “Shelf labeling on its own is unlikely to significantly influence consumer behaviors” [75].

Of specific interest is the large-scale randomized trial that compared purchases of 1266 food products from 4 categories in 60 real French supermarkets which displayed different FOPNLs [28]. The effect sizes were 17 times smaller on average than those found in comparable laboratory studies, and especially in the above quoted study [56]. The most effective nutrition label, Nutri-Score, increased the purchases of foods in the top third of their category nutrition-wise by 14%, but had no impact on the purchases of foods with medium or low nutrition quality. Therefore, Nutri-Score only improved by 2.5%, i.e., 0.142 FSA points the nutritional quality of the basket of labeled foods purchased, to be compared with the 2.65 points improvement seen in the laboratory study [56].

Thus, real-life studies more often have mixed results about the effectiveness of FOPNLs on food purchases than laboratory-set ones; furthermore, they observed effects of a much lower magnitude, which are also more susceptible to be affected by multiple confounding factors All this calls for caution when interpreting findings from laboratory-set studies which may not be generalizable to real-life situations, and also calls for more “real-life” studies which today represent only 20% of all studies.

#### 3.1.3. Is the Effect of FOPNL Dependent on Food Category?

Another important difference across studies concerns the foods and food categories they considered and monitored. This relates to the number of foods and categories of foods that are displaying a FOPNL during the experiment and to whether the nutritional quality outcome concerns only the foods bearing a label (do people move to food choices bearing a “healthier” logo?) or the whole shopping basket, which may contain labelled and unlabeled foods in varying ratio [34,41,46,56,66,75], Studies were very diverse in this respect: some restricted their investigation to a limited number of foods, such as nine products often consumed in Brazil [64] or to products within a same category, such as beverages (sodas, milk, fruit juices, bottled water, etc.) or within an eating occasion such as snacks (e.g., potato chips, candy, cookies, apple, carrot, processed cheese, meat snacks, etc.) [62]. Other authors monitored purchase/selection behaviors across different versions of various nutritional quality of similar foods, such as cookies [71,72], pizzas [30,74], or dairy products [63,72].

Study findings suggest that the effect of a FOPNL depends on the type of foods, although it remains unclear which foods would be more ”sensitive” to FOPNLs. When several foods belonging to different categories were compared within the same study, the magnitude or even the significance of the effect indeed varied across foods. For example, in a sample of Peruvian students, WS impacted the choice among 3 sugar-sweetened beverages with the healthiest version being chosen more often (+27.4%) and the less healthy version less often (−25.7%) as compared to no label group. But WS had no effect on purchase/selection of crackers of varying nutritional quality [23]. Differences in the effect of Nutri-Score were also seen between pizzas, cakes, or breakfast cereals in laboratory-type studies [32,63,83] while, in a real-life study across the whole supermarket food offer, the Nutri-Score was found to have different impacts, according to categories. Impacts were favorable (with either an increase in healthier and/or decrease in less healthy food sales) for 17 out of 58 food categories, representing about 29% of total food sales and including in particular vegetables, fruits, dairy products, and confectionery. But the impact of the Nutri-Score was unfavorable (with either a decrease in healthier and/or increase in less healthy food sales) for 16 other food categories, representing 24% of total food sales and including bread and bakery products [75].

Results also suggest that there is apparently no link between the a priori perceived healthiness of a food category and the performance of Nutri-Score. Conversely, it had been found that the number of WS impacted on intention of purchase of indulgent foods (ketchup and ice cream), but not on those said to be perceived as more staple or “utilitarian” (margarine and cereal bar) products [51]. The relevance of the perceived healthiness of a food in the performance of FOPNLs has been addressed in a recent lab-set study, where participants were given 5 USD to purchase one of 20 beverages. No FOPNL showed any effect, except for plain milk, which was purchased in 5.5% of cases when bearing the HSR logo, vs in 7.7% of cases when no FOPNL was displayed, which can be seen as an unwanted deterioration of choices with HSR. [62].

Several other food attributes may interact with the expected effect of a FOPNL and only a few have been studied. Sensory expectations are likely to play a major role, as suggested by the reaction of Brazilian adults and children who were about to select sugar-reduced versions of beverages (grape nectar and chocolate flavored milk), following the indications of WS or MTL, until they tasted the products: final choices were conditioned by the sensory characteristics and the product without sugar reduction were the most frequently selected, suggesting that consumers’ hedonic experience overrode the effect of FOPNL [20]. Brand loyalty may also interfere with the reaction to a FOPNL. Indeed, it is well known that a positive attitude towards a brand influences a customer’s purchase intention and this may create a conflict when a product from an appreciated brand displays an indication of low nutritional quality. When Spanish adults were exposed to yoghurts from their favorite brands, the presence of Nutri-Score did not modify the relation between brand attitude and food purchase attitude: familiarity and trust towards the brand protect the product from being evaluated as unhealthy based on the Nutri-Score letter [78]. These studies need to be confirmed, but they suggest that sensory expectations or brand loyalty could have stronger effects than FOPNL in food selections/purchases

It seems that the category or the characteristics of the labelled food may interact with the effect of a FOPNL on purchase/selection by consumers, yet in a still non-understood way. Available studies have included a large number of foods or categories but their attributes, such as cost, taste, role in a meal/diet, serving size, etc., together with the way they are perceived by consumers have not been systematically addressed as potential explanatory factors. Future studies should better inform this relationship, in order to preferentially display FOPNL on the products for which consumers need help for purchase decision.

#### 3.1.4. What Is the Durability of FOPNL Influence on Food Purchase/Selection?

From a public health perspective, it is critical that the expected favorable effect of FOPNLs on the purchasing behavior of consumers is maintained over time. Demonstration of a long-lasting effect on FOPNL is currently lacking, and studies are mostly cross-sectional, with a single assessment of the outcome or with assessment performed in a time period that is too short to conclude to a sustainable change in consumer behavior. As a matter of fact, national health policies that recommend a FOPNL are quite recent. Furthermore, FOPNLs are voluntary in most countries, except for WS in Latin America, which adds complexity to the investigation of their effectiveness on purchasing behavior over time. Indeed, the number of products that displayed a FOPNL is continuously evolving, usually with an observed trend towards an increase in the number of labelled products [21,22,49]. Experimental studies with several replications in the same subjects, or empirical studies led in the same setting (equivalent number of labelled products, similar food categories.) including several assessments over a long enough time period would be required to evaluate whether FOPNLs may have long-lasting effects and may thus have a real impact on nutrition and health.

Such studies are today not available, to the best of our knowledge, except one report from New-Zealand, which suggested that the effect of HSR-labeling in driving choices towards healthier breakfast cereals, which was significant just after the implementation of the HSR, strongly decreased after one year, despite a large communication campaign [38]. Of interest regarding the sustainability of the observed effect is also a quasi-experimental study which investigated the impact of the Nutri-Score on the nutritional quality of meals of subjects frequenting staff cafeterias. Meals were recorded for 6 weeks before and 7 weeks after implementation of the Nutri-Score. Mixed effects models showed that the intervention had a significant immediate beneficial impact on the overall nutritional quality of meals, which however decreased over time, suggesting that the improvement of food choices is not maintained on the long term. Surprisingly when the amounts of energy and nutrients were considered rather than the overall quality of meals, the initial quantitative effects on nutrient intake were unfavourable, with higher reported energy and all nutrients except salt. However, there was a subsequent significant decrease over time, except for salt where the opposite trends were observed. An immediate reduction in salt was followed by a significant increase over time. It might be that the presence of the Nutri-Score has initially created a “health halo”, which prompted consumers to increase their intakes of healthier foods, but disappeared after some time. This may also suggest that consumer’s interest and attention vanish overtime and that FOPL have no sustained effect on overall meal quality over time’ [69].

#### 3.1.5. Does the Influence of FOPNL on Food Purchase/Selection Depend on the Consumer?

Study participants were mostly recruited in the general population. Except when quota sampling was applied for the recruitment [12,30,31,35,49,52,55,61,68,79,84], females were usually over-represented, and this may be a bias as women might be more reactive to FOPNLs than men [23], although some studies did not find any gender effect [68]. The behavior of adults (from young adulthood to early senior age), who are most likely to be in charge of the household purchases, was most often investigated but few studies included or were focused on adolescents [59,71] or children [60,85]. When young people are specifically targeted, there are indications that their choices can be improved by a FOPNL, such as for French children choosing better quality foods and beverages for their afternoon snack when Nutri-Score was displayed [60], or in Peruvian adolescents who rather selected foods with no WS compared to foods with 1 or 2 black WS [71].

It is well known that current food environments are creating inequities, and FOPNL should be a tool able to reach the populations who are the most concerned by improving their food choices towards healthier products [86]. No study has directly addressed this concern, although analyses were sometimes stratified by sociodemographic characteristics of participants (income and education). These latest studies provided inconsistent results regarding a possible role of income or education in moderating the impact of the studied FOPNLs. For example, in Mexican adults exposed to MTL, WSs or no FOPNL, the nutritional quality of the shopping cart tended to be lower among those with low income, education and nutrition knowledge levels, whatever the labelling condition [41], an observation also made in Ecuador about carbonated soft drinks purchases [12]. Conversely, in a sample of young Peruvian students, there was evidence that MTL labels impact more on food choices among individuals with limited nutritional knowledge [23]. In Australia, income and education did not mediate the willingness to pay more for a box of cookies displaying the HSR vs no FOPNL [55] and were not either influencing the intention to purchase healthy beverages bearing a HSR [68].

Overall, it seems that age, income, or education have little importance in the reaction a consumer has to FOPNL, which may suggest that their expected effect on food selection/purchase should be the same whatever the socio-economic status of the population. A single study, to the best of our knowledge, has tested the effect of a FOPNL on a low-income French population (2016 income: 1200 € (approx. 1090 USD) and found no effect of NS compared to a no FOPNL condition on the healthiness of food selections [37,58]. More research is critically needed in vulnerable populations who are the most likely to benefit from improved food choices.

#### 3.1.6. Does the Display of FOPNLs Translate into Healthier Eating Habits?

One of the goals of FOPNLs is that, through the selection and purchase of healthier foods, consumers adopt healthier consumption habits. There is ample evidence from consumer research that knowing what is healthier does not directly translate into healthier behaviors. An interdisciplinary meta-analysis of the FOPNL effects on consumer behavior found that their favorable effects on consumers’ choice of healthier options, were much weaker than for the identification of the healthier options [87]. Very few studies have gone beyond the selection or purchase process and it is thus difficult to check whether or not this goal is achieved. This has been addressed in the former European JRC report [18] which concluded that “there is no available empirical evidence to link FOPL in general or any FOP scheme in particular directly with concrete changes in food intake. As mentioned earlier, proving this causal link is a daunting task”. The evidence is still very weak as no study has been identified that would assess real food intakes in relation to the FOPNL of interest. One study however has tested the effect of a green low-fat label, a red high-fat label (broadly comparable to the black WS) or no label on the intake of popcorn by UK subjects watching a movie; no effect of this FOPNL was seen on consumption [88].

An intermediate approach can be to examine whether the display of FOPNL could influence the selected portion size of foods (reducing it for lower quality foods and/or increasing it for foods of higher nutritional quality). Two studies asked participants to indicate the portion size they believed should be eaten of each food on a single occasion; one food displayed a FOPNL and one did not. Results, which do not correspond to actual consumption of the product, did not provide a clear conclusion: the presence of Nutri-Score appeared to reduce the selected portion size for sweet biscuits, sweet spreads and cheese, as did MTL [19]; however Nutri-Score and MTL had no effect on the portion size of pizza or cookies. To the opposite, displaying HSR led to a lowering of the portion size of pizza, but not of cookies [50].

There is a significant research gap in this area; although it can be expected that purchased food will ultimately be consumed, the impact of FOPNL on actual consumption has not been investigated, and its magnitude and thus its relevance in a Public Health perspective is unknown. Dedicated studies are required, to help address the individual level of consumption, as purchases concern the household.

#### 3.1.7. Are Some FOPNLs More Effective Than Others?

Although this review does not consider all available FOPNLs, differences in the effectiveness of the four selected schemes are of interest. Such comparisons can only be attempted from studies which included several of these FOPNL in the same experimental design. Seven studies compared the four FOPNLs [30,32,34,52,62,63,66], five having been carried out by or with the research team who developed and promotes the Nutri-Score [30,32,35,52,66]. These five studies were web-based experimental studies with the same design, comparing selection of food products in 20 different countries worldwide. The performance of the different FOPNLs vs selection of healthier products within a food category was compared by measuring how a FOPNL either improves, deteriorates or has no effect relatively to a control, non-interpretative label. Authors consistently concluded that the Nutri-Score was the most efficient FOPNLs. This superiority was however often very limited, such as when tested in Portugal: when compared to a non-interpretative label, the adjusted odd ratio for healthier choices was 1.98, 1.95; 1.94 and 1.4 for Nutri-Score, MTL, HSR and WS, respectively [66] Furthermore, it seems that the performance of Nutri-Score, both absolutely and relatively to other labels, depends on the country. In a European study carried out in 12 countries (sample size around 1000 in each country), the overall performance of Nutri-Score, then of MTL outpaced those of HSR and WS. When analyses were performed by country, the improvement of the nutritional quality of food choices with Nutri-Score was solely significant in France. In other countries, only non-significant trends were typically observed of a higher performance of Nutri-Score over other FOPNLs, whose performance varied by country [32].

Other studies failed to show a superiority of Nutri-Score vs the other FOPNL: in Morocco, all systems were equally inefficient in improving the healthiness of food choices [63]. In Canada, no significant differences in purchases were observed neither vs a no label control or across the labeling conditions for fruit juice, chocolate milk, or cheese snack products; HSR tended to encourage best purchases of products with certain positive nutritional attributes, while WS and MTL appeared to more effectively discourage purchases of products contributing to nutrients of public health concern [62].

Other studies have compared three [36,39,53,56,73] or two [20,27,34,41,42,50,59,79] FOPNLs and their findings do not provide convincing evidence of the superiority of one vs the others, as rankings, usually not statistically significant, varies according to comparisons and to the designs and characteristics of studies, such as the type of foods. Finally, a recent network meta-analysis attempted to compare the effects of Nutri-Score, MTL and WS and found, without providing a ranking, that MTL and WS were associated with an increased probability of selecting more healthful products, while Nutri-Score appeared effective in reducing consumers’ probability of selecting less healthful products [89].

Importantly, this review showed that there is no “gold standard” among FOPNLs. The key characteristics that would be needed for a FOPNL to be efficient are not universally established and may depend on the context and Public Health objectives.

#### 3.1.8. Can FOPNL Have Unexpected Consequences on Consumer Behaviors?

Some findings suggest that the display of FOPNL may have unplanned consequences, for example on some individuals for whom sensory expectations remain the first driver of food purchases and who anticipate that unhealthy products will be tastier while healthy ones will be less rewarding [87]. This may explain why the presence of a FOPNL may lead to lower quality food choices as sometimes observed.

The presence of a FOPNL in itself, compared to no label, may generate unexpected reactions. In some cases, it may create a kind of “health halo”, that will increase selection independently from the actual information carried out by the FOPNL, with no global improvement in the nutritional quality of food choices. This is more likely to occur when FOPNL are displayed on a voluntary basis [18]. FOPNL may also divert attention from the back-of-pack nutrition facts panel [87], leading paradoxically to a reduced nutritional awareness [49].

These limited, largely anecdotal indications that FOPNLs may have negative consequences deserve dedicated studies to better measure their real impact.

### 3.2. Are FOPNL Impacting Diet Quality and Nutrition Outcomes?

In some studies, consumers’ food purchases following exposure to FOPNL were recalculated into purchases of nutrients, with a focus on saturated fats, sodium, and sugars. In a recent meta-analysis, which identified only 8 experimental studies, sugar and sodium contents of purchases were lower for groups exposed to any FOPNL (including, but not limited to the four FOPNL of interest in this review) versus no-FOPL, with a non-significant trend for lower energy and saturated fat [90]. Based on daily intakes of 2000 kcal and 100 g sugar, theoretical models based on the very optimistic assumptions that all monthly food purchases would be as favorably affected, suggest that energy would decrease by <1% and sugars by <4% [90]. Real-purchase data from more than 20,000 British households were collected during a 3-year period which included the year of the UK Food Standard Agency recommendation for retailers to adopt MTL on all store-brand products within 7 food categories (ready meals, burgers, pies, breaded/coated meats, pizzas, sandwiches, and cereals). This study reported reductions of the total monthly intakes of calories (588 kcal), saturated fats (−13.7 g), sugars (−6.9 g) and sodium (−0.8 mg) from store-brand MTL labelled food purchases, showing a positive response from households to MTL, which however remains very limited, with daily reductions not exceeding 20 kcal or 230 mg sugars [33].

Fewer studies addressed the impact of FOPNLs on the overall nutritional quality of total food purchases. For example, the Healthy Eating Index (HEI) of the whole basket was improved when Singaporean participants made their online purchases from a set of 4000 foods and beverages displaying a FOPNL (Nutri-Score or MTL) vs a set without any FOPNL. The HEI in the no-FOPNL condition was 41.8 (95% CI: 40.7–42.9) and was increased by 1.09 ± 0.53 and 1.16 ± 0.53 when Nutri-Score or MTL were displayed [34].

These studies confirm the low magnitude of the effects of FOPNLs, even in artificially maximized hypotheses. Of note, studies have so far only addressed the potential impact of FOPNL on nutrients to limit, but not on shortfall nutrients, such as iron, calcium, vitamin D or fibers, whether or not the latter are included in the FOPNL scheme, such as for fibers in Nutri-Score.

### 3.3. Are FOPNL Affecting Modeled Health Outcomes?

Improving consumer’s health is the ultimate goal of any FOPNL. However, as stated in the JRC report in 2020: “Considering the lack of available real-life evidence, and given the difficulty to set up such studies, no definitive conclusions can be drawn at this point regarding the effect of FOP nutrition labels on diet and health” [18]. Eight studies, published after mid 2018 were identified that dealt with this topic and all were modelling studies, addressing mortality, cancer and overweight/obesity risks. Some assessed the quality of diets of individuals participating in longitudinal studies, by the means of nutrient profiling scheme underlying a FOPNL and evaluated its association with the health benefits monitored on the long-term [25,26,29,31,45,61]. Other studies undertook different modelizations, transferring the improvements observed in nutrient intakes (see above) to dietary data from participants of large cross-sectional studies. Finally, a model designed to estimate the number of cases (deaths, CVD, obesity, etc.) associated with nutrient intakes was applied to both theoretical and actual nutrient intakes. The difference between both estimates corresponded to the number of cases that could be averted with the introduction of FOPNL [43,54].

All these modeling studies found favorable improvements in health, with impressive figures such as 8435 cardiovascular deaths saved per year if Canadians avoided foods labelled with red traffic lights [40]. The reduction in energy intakes would by itself save 10,490 deaths [43], which should be put in perspective with the findings that decreases in energy that could be actually due to MTL in the UK were less than 600 kcal/month or 20 kcal/day [33]. Such favorable results are not surprising, considering the very optimistic assumptions on which models are based (e.g., all consumers responding as expected to FOPNL by always choosing the best option, mandatory FOPNL on all food products, no modification of the diet as compared with baseline assessment, etc.). The very significant impact of diet, and especially of excessive energy, sugar, sodium and saturated fat intakes is well known [86] and any simulation that would decrease such excessive intake would end in lowering the incidence of these diseases.

While illustrating the power of a healthier diet in reducing morbidity and mortality from non-communicable diseases, a well-known feature and the reason-why of current dietary guidelines, these studies do not really show how FOPNL contribute to this desired situation. More investigations are needed, but will be difficult to implement as they should be long-term real-life studies, with potential methodological drawbacks and numerous confounding factors.

### 3.4. Are FOPNLs Promoting Product Reformulation by Food and Beverage Manufacturers?

As highlighted in the JRC EU report, “the goal of the regulator to foster the consumption of healthier diets may be achieved also through the food supply side” and “as long as FOPNLs may affect consumer’s choices, producers have an incentive to adapt the content of their products to the requirements needed to obtain a good nutritional rating” [18]. Although their number has increased in the last few years, studies of this topic remain few. Indeed, the favorite design consists in comparing the nutritional content/quality of food before, then after, the implementation of a FOPNL: this implies that the FOPNL has been present on the market for several months, and preferably years.

Eight studies dealing, more or less directly, with the impact of FOPNL on the food offer have been published since mid-2018, five of them investigating the effect of HSR on the Australian/New Zealand [47] market [21,22,57,76,80], and two dedicated to WS in Chile [47,48], reflecting their longer presence on the market. Results were not fully consistent, perhaps because of different methodologies: different food categories were used across studies, and new product launches were not treated in a similar manner.

For example, food nutritional labeling declarations from 70% of the most consumed packaged foods in Chile were analyzed in 2013 and 2019, i.e., before and after the implementation of WS. Total sugar was significantly reduced (−15%), but trends in reduction in sodium, energy or saturated fats did not reach significance [47]. Reformulation associated with HSR adoption was investigated in New-Zealand over seven years (2013–2019) and in Australia over five years (2014–2018), on more than 58,000 foods covering 14 categories. In Australia, the reformulation of HSR-labeled products resulted, respectively, in decreases of 0.5% and 1.4% in sodium and energy contents, but no change was seen for SFA and sugars. Unexpectedly, the fiber content of HSR-labelled products was decreased by 1.6%. Different results were found in New Zealand, where the reformulation of HSR-labelled products resulted in decreases of 4.0% and 2.3% in sodium and sugar contents, but no change was seen for SFA and energy. However, the fiber content of HSR-labelled products was increased by 1.9% [22]. When examining changes in foods marketed towards children in Australia between 2013 and 2016, no change was seen in energy content, but a decrease was observed in the sodium content (−20.2 mg/100 g), across the 100 foods that were available at both times [21]. The effect of HSR adoption was significant in cereal-based products, with decreases in sugar and sodium contents, but not in fruit-based or in dairy products over that period, suggesting that some categories, may be easier to improve [21].

Other studies focused on the number of products displaying the FOPNL. When this concerns a mandatory FOPNL flagging the low nutritional quality products, such as the WS, a decrease in the number of products displaying it is likely to indicate an improvement of the food offer, and this has been the conclusion of two Chilean studies which found less products display a “High in” WS for energy, sugars, SFA and salt following WS implementation [44,45]. For other voluntary FOPNLs (MTL, Nutri-Score and HSR), the observed increase in the number of products displaying a FOPNL [21,22] cannot be associated with an improvement of the food.

Because FOPNLs are susceptible to influence consumers’ purchases, food manufacturers may tend to improve the quality of their products in order to get a competitive edge. Indeed, findings overall suggest a slight beneficial effect of FOPNL on food reformulation, but evidence is still limited. A broader implementation of the FOPNL will be followed by more studies that would provide more grounds for such a conclusion. However the effectiveness of the FOPNL in this process may be modified by concurrent public health campaigns, marketing strategies and new product launches from food and beverage manufacturers.

## 4. Discussion

This review gathered studies investigating the efficacy of four selected FOPNL (MTL, Nutri-Score, HSR and WS) on the food selection and/or purchase behavior of consumers and on the actual improvement of the nutritional quality of the packaged food offer. Although trends, sometimes statistically significant, can be seen in some studies towards an improvement of food choices and food offerings, these improvements are small in magnitude and their real impact on consumer’s health remains elusive. Thus, in spite of a growing number of studies, the scientific evidence today is still limited and insufficient to clearly demonstrate that the use of any of these four systems has a positive effect on the healthiness of the consumer’s food purchases in real life or is incentivizing food operators to improve the nutritional quality of their products. Overall, the favorable statement that FOPNL are effective needs to be nuanced and contextualized, in relation to study design and characteristics.

### 4.1. Comparison with Other Reviews

This conclusion is in line with other recent reviews on the same topic, which concluded on the limited efficacy of FOPNL vs consumers’ purchases. For Ikonen et al. ‘*although FOP labels help consumers to identify healthier products, their ability to nudge consumers toward healthier choices is more limited*’ [87]. An et al. conclude that ‘*findings on the effectiveness of FOP nutrition labels in ‘nudging’ consumers toward healthier food purchases remain mixed and inconclusive*’ [91]. This is also the conclusion of an update of the 2020 JRC report [92], covering the literature published up to February 2021, which states that ‘*the available evidence on actual shopping behavior suggests a small beneficial effect of FOPNL on ‘on-the-spot’ purchasing*’. Other reviews have however concluded differently, such as the network meta-analysis of Song et al., which states that ‘*colour-coded labels and warnings appeared effective in nudging consumers’ behaviour towards more healthful products by changing the healthfulness perception and eliciting negative emotions*’ [89], or the one of Croker et al. who underlined the lower purchased amounts of sugar and sodium [90]. This discrepancy in the final interpretations of the same original studies probably lies in the importance given to the methodological drawbacks of these studies, which are widely recognized, as well as to the magnitude of the observed effects. Methodological limitations include the high risk of bias which has been detected in more than half of the studies [89]; the high proportion of laboratory or on-line studies, which report intention rather than genuine purchases; the limited number of foods or food categories, and more generally the variability in designs and conditions, that resulted in high heterogeneities in meta-analyses [87,89,90].

For some authors, these limitations are preventing conclusions that FOPNLs have demonstrated favorable effects on consumer purchases. This is exemplified in an evaluation of the Nutri-Score system along the reasoning for scientific substantiation of claims in the EU, as undertaken by the European Food Safety Agency (EFSA), following rigorous scientific standards. Based on this EFSA approach, there is insufficient evidence to support the claim that NS a results in an increased purchase of healthier foods by consumers’ since a cause-and-effect relationship could not be established [93]. For others authors, these limitations do not preclude the use of FOPNLs as promising tools in directing consumers’ food choice and encouraging reformulation in the food industry [89].

### 4.2. Differences and Specificities across FOPNLs

This review has considered four FOPNLs, which have been implemented at various degrees in different geographies and which have attracted the most of FOPNL research. However, the diversity of studies and findings, together with the limited number of comparative studies make difficult, and even not possible, to detect whether one of these four system has led to better outcomes than the other ones. There is no “gold standard” among them and it does not appear possible to establish any type of efficacy ranking and the basis of the available studies. Familiarity one has with a FOPNL is an additional confounder, as suggested by a comparative study where Nutri-Score, HSR and MTL were assessed for their performance in changing consumers’ ability to correctly rank products between no label and labelling conditions. NS performed better than the other ones in France, MTL in the UK and HSR in Australia, suggesting that the familiarity one has with the system is a key factor in the measured efficiency [92]. Comparisons among FOPNLs is also complexified by the multiple differences across them: some are nutrient-specific (WS, MTL), others are summary indicators (Nutri-Score, HSR), some are colored (Nutri-Score, MTL), others are neutral (WS, HSR), some are mandatory and injunctive (WS), others are voluntary and informative (Nutri-Score, HSR, MTL), with all these features being likely to influence effects. Some authors have however expressed preferences, in spite of weak scientific support. WS is sometimes thought to be more effective because it includes a “stop” dimension [93]. Others are strongly promoting Nutri-Score as the best option in Europe [94].

### 4.3. Research Gaps

Most of the reviewed studies pointed to research gaps that needed to be addressed. Chief among those were firstly the lack of well-designed empirical studies to better estimate the “real-life” effects of the introduction of FOPNLs on consumers’ purchases. Such studies should ideally consider the quality of the whole diet, or at least of the whole food basket, in order to monitor the possible displacement of food purchases towards less healthy foods that may be unpackaged or without a FOPNL. Careful attention should also be given to potential confounding factors, such as promotion or communication campaigns.

Also cited was the absence of longitudinal studies of sufficient duration, which are required to assess whether the acute effect that may be observed in short- or medium-term studies will be maintained over time. In addition, there was limited information on the individual factors, especially socio-economic ones, that may interfere with the effect of FOPNLs. Indeed, improvements in food choices are especially needed in the less educated and lower socio-economic classes and ensuring that FOPNLs are efficient in these group of populations is a pre-requisite. Furthermore, large-scale independent evaluations are missing to assess the impact of FOPNLs on (re)formulation of food products by the industry, which can only be carried out when a FOPNL has been implemented for a sufficient duration.

There were concerns about the potential unintended consequences of FOPNLs which should be considered, both systematically in future studies, and also through dedicated experiments aimed at exploring specific questions, such as the reality of a “health halo” or of inadequate changes in purchasing behaviors. Paying attention to the evolution of the nutritional composition of the food offering is also of relevance; indeed, industrial stakeholders will be incentivized to improve their products for the nutrients that are taken into account in FOPNL and may provide less attention to other ones which are nevertheless important. Most FOPNLs today are triggering decrease of energy, saturated fats, sodium and sugars and there is very little incentive to increase favorable nutrients, which are often shortfall ones, such as calcium, vitamin D, polyunsaturated fatty acids, or dietary fibers, or to promote wholesome ingredients such as wholegrains, fruits, vegetables, or legumes. Knowing that diets low in these favorable nutrients or components are more strongly associated with non-communicable diseases than diets high in unfavorable nutrients (except for sodium) [95], it is of primary importance to check that FOPNL do not inadvertently lower their supply and intakes.

Finally, other relevant research is needed, such as those which compare costs and benefits of the implementation of FOPNL with those other nutrition policies (nutrition education, reformulation program, marketing restrictions, taxes, etc.) or which dig more in psychological dimensions that might support the effect of FOPNLs on consumers’ behaviors, in order to potentially improve the features of a most efficient FOPNL.

The 2022 update of the JRC report [92] also highlights most of these research gaps, that more real-life evidence would help corroborate the magnitude and sustainability of the effect of FOPNL schemes on actual purchasing behavior, nutrient intakes, and the extent to which FOPNL schemes may lead to improvements of the overall diet and health. Long-term real-life studies in purchase settings would indeed provide the best scientific evidence, but their implementation will be difficult and expensive. Researchers might however better exploit the new means given by on-line grocery shopping, where real, monetized purchases occur. Furthermore, thanks to the loyalty cards, consumers could be followed over time and characterized in socio-demographic terms. Alternatively, as FOPNLs are currently being implemented in several regions/areas, follow-up programs could be conceived in advance, that would allow to monitor changes connected to these implementations. This may also provide information about the effect of FOPNLs on the nutritional quality of the food offer, an area where partnerships with manufacturers and retailers would be efficient.

## 5. Conclusions

Improving the quality of the diet is one of the major challenges our societies are facing and all possible means should be considered to achieve this goal. FOPNLs are relatively new tools that are being developed for this purpose and are giving rise to a growing volume of scientific research, which today provides some, but insufficient evidence that FOPNLs can lead to meaningful improvements in consumers’ behaviors and nutritional quality of the packaged food offer; this is the case for the four FOPNLs which have been considered in this review. This does not mean that FOPNLs should not be part of the tool box, but that a significant amount of research is required both to improve the characteristics of FOPNLs and their conditions of use and to appropriately measure their possible contribution to the overall effort that remains needed.

## Figures and Tables

**Table 1:** Key features and information on the current use of the four selected FOPNLs.

	Key Features	Current Use
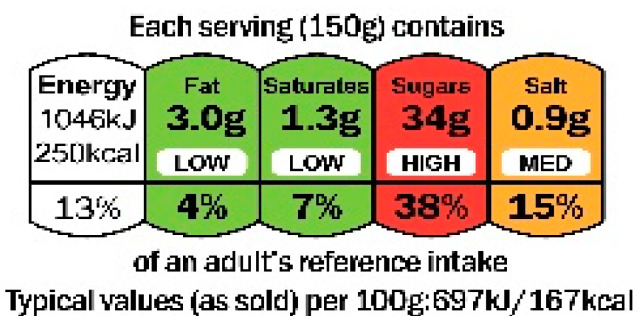 Traffic Lights [10]	Nutrition information (energy plus fat, SFA (saturated fatty acids), sugars, and salt) in g and as % of daily reference intake. Color coding indicating low (green), medium (amber), and high (red) levels of the nutrients stated. Portion as reference base for numerical information; 100 g or mL as reference base for color coding & additional energy information.	UK authorities recommended MTL in 2013. A significant number of businesses, and all major retailers are displaying the MTL on all, or a selection of products [11]. A slightly adapted version is in use in Ecuador since 2014 [12]
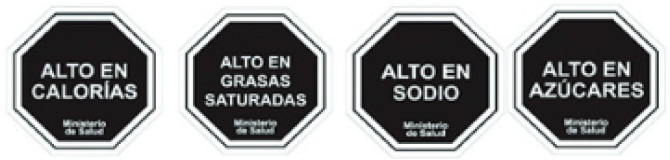 Warning Signs [6]	Black WS on foods high in energy, sugar, sodium, SFA, with differences in thresholds and reference basis (100 g/mL or serving), depending on the country.	Currently used in Chile, Peru, Uruguay, Mexico, Columbia, and Argentina, and mandatory in all instances [13]
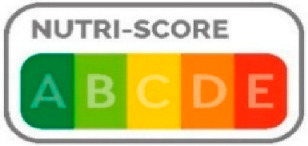 Nutri-Score [14]	Graphic scale with 5 classes (expressed by a color and a letter), based on the food content of energy, sugars, SFA, sodium, ‘fruit, vegetables, pulses, and nuts’, fiber, and protein. Nutritional quality decreases from green “A” to red ”E”. Algorithm based on UK Food Standards Agency (FSA) Nutrient Profiling system. Reference base for the score calculation is 100 g or mL.	Supported by the government in Belgium, France, Germany, Luxemburg, Netherlands, Spain and Switzerland. In France in 2021, 57% of sales of packaged foods displayed the NS logo [15]).
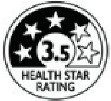 Health Star Rating [16]	Points-based system that attributes a summary score between 0.5 and 5 stars, from poorest to best nutrient profile. Contents of the food in energy, fat, sugar, sodium, fibers, proteins, fruits & vegetables are computed, using 100 g or mL as the reference base. The score is converted into stars using food group-specific conversion keys. May be complemented with quantitative energy and nutrient content information, per 100 g/mL, or pack	In use in Australia and New Zealand, on a voluntary basis. In 2022, present on over 10,300 packaged foods [17].

**Table 2:** Number of original studies sorted by FOPNL and by outcome. Note that a same study can address several FOPNL and/or several outcomes.

	Nutri-Score	Multiple Traffic Lights	Warning Signs	Health Star Rating	Nb of Publications
Outcome					
Purchase or selection	28	28	24	19	52
Nutrition & health	10	7	4	4	16
Reformulation	0	1	2	5	8
Nb of publications	34	30	27	28	

## Supplementary Materials

The following are available online at https://www.mdpi.com/article/10.3390/nu15010205/s1, Table S1: Data extraction and analysis of the 65 experimental studies addressing the impact of 4 FOPNLs (Nutri-Score, Multiple Traffic Lightin, Health Star rating, and Warning signs) on consumers’ selection and purchases of foods, on diet quality and on modelled health outcomes.
